# Spatially specific vs. unspecific disruption of visual orientation perception using chronometric pre-stimulus TMS

**DOI:** 10.3389/fnbeh.2015.00005

**Published:** 2015-01-30

**Authors:** Tom A. de Graaf, Felix Duecker, Martin H. P. Fernholz, Alexander T. Sack

**Affiliations:** ^1^Faculty of Psychology and Neuroscience, Department of Cognitive Neuroscience, Maastricht UniversityMaastricht, Netherlands; ^2^Maastricht Brain Imaging CentreMaastricht, Netherlands

**Keywords:** TMS, vision, awareness, orientation processing, masking, suppression, biasing, state-dependent

## Abstract

Transcranial magnetic stimulation (TMS) over occipital cortex can impair visual processing. Such “TMS masking” has repeatedly been shown at several stimulus onset asynchronies (SOAs), with TMS pulses generally applied after the onset of a visual stimulus. Following increased interest in the neuronal state-dependency of visual processing, we recently explored the efficacy of TMS at “negative SOAs”, when no visual processing can yet occur. We could reveal pre-stimulus TMS disruption, with results moreover hinting at two separate mechanisms in occipital cortex biasing subsequent orientation perception. Here we extended this work, including a chronometric design to map the temporal dynamics of spatially specific and unspecific mechanisms of state-dependent visual processing, while moreover controlling for TMS-induced pupil covering. TMS pulses applied 60–40 ms prior to a visual stimulus decreased orientation processing independent of stimulus location, while a local suppressive effect was found for TMS applied 30–10 ms pre-stimulus. These results contribute to our understanding of spatiotemporal mechanisms in occipital cortex underlying the state-dependency of visual processing, providing a basis for future work to link pre-stimulus TMS suppression effects to other known visual biasing mechanisms.

## Introduction

Transcranial magnetic stimulation (TMS) is a method of non-invasive brain stimulation used to enhance (Duecker et al., 2014) or disrupt (Pascual-Leone et al., 2000) performance on perceptual, cognitive, or behavioral tasks. The application of occipital TMS to disrupt visual processing has since its establishment (Amassian et al., 1989) been replicated many times (Beckers and Hömberg, 1991; Kammer and Nusseck, 1998; Kastner et al., 1998; Kammer, 1999; Kammer et al., 2005a,b). It should be no surprise that TMS pulses administered to early visual cortex (EVC) after the presentation of visual stimuli can interfere with visual processing. After all, taking into account retino-cortical transmission delays, EVC should process the visual properties several tens of milliseconds after stimulus onset (Baseler and Sutter, 1997; Di Russo et al., 2002; Vanni et al., 2004). And indeed, magnetic pulses can disrupt online cortical processing around 70–130 ms (for reviews, see Kammer, 2007; de Graaf et al., 2014).

Interestingly, EVC appears to be functionally relevant at multiple stimulus onset asynchronies (SOAs). Aside from the classical 70–130 ms window, TMS masking has been reported in earlier SOAs around 20–40 ms (Corthout et al., 1999a,b, 2002, 2003; Paulus et al., 1999; Kammer et al., 2005b) and later SOAs around 200 ms and even later (Juan and Walsh, 2003; Heinen et al., 2005; Camprodon et al., 2010; Chambers et al., 2013; Allen et al., 2014). But as long as TMS pulses are applied after the presentation of the visual target stimulus, these suppression effects can be explained by disruption of functionally relevant online processing, whether by decreasing signal or by increasing noise (Miniussi et al., 2013). Such an explanation becomes more difficult if TMS suppresses stimuli that are presented only afterwards.

Yet early reports suggested that indeed there might be a second class of TMS suppression effects, with TMS pulses applied before the onset of a visual stimulus, at “negative SOAs” (Corthout et al., 1999a,b, 2000, 2003). Single TMS pulses are not “supposed” to have effects that last for hundreds of milliseconds (although see Moliadze et al., 2003). And at any rate, the same cortical mechanism as in the “classical masking window” around +100 ms cannot be disrupted by pre-stimulus TMS, since not all intervening SOAs are equally sensitive to TMS disruption (Corthout et al., 1999a,b; Breitmeyer et al., 2004).

An ongoing discussion concerns the nature of pre-stimulus TMS suppression effects; specifically whether these effects reflect neural suppression/biasing or simply indirect non-specific TMS effects (Duecker and Sack, 2013; Duecker et al., 2013) such as eye blinks (Corthout et al., 2000, 2011; Jacobs et al., 2012b). Moreover, the question is relevant in light of increased interest in state-dependent visual processing (Britz and Michel, 2011). Fascinating recent studies related pre-stimulus mechanisms to subsequent perceptual/attentional success (Thut et al., 2006; van Dijk et al., 2008; Busch et al., 2009; Mathewson et al., 2009; Britz et al., 2014; Jaegle and Ro, 2014). Since TMS can probe the functional relevance of brain mechanisms, moreover with high temporal resolution, pre-stimulus TMS modulation of subsequent visual processing may contribute much to this field.

After pioneering work by Corthout et al. (1999a,b, 2003), we recently investigated pre-stimulus TMS suppression effects in a series of studies. Discrimination of complex arrow stimuli was reduced by occipital pre-stimulus TMS, an effect not observed for SHAM or real vertex-TMS (Jacobs et al., 2012a) and not attributable to eye blinks (Jacobs et al., 2012b). This effect was generally strongest with TMS pulses applied around 70–40 ms prior to visual stimulus onset. Interestingly, we recently showed that this visual suppression effect was spatially unspecific or “global”, by which we meant that it was independent of visual field location as opposed to the traditional spatially specific post-stimulus TMS masking effects, even though we again excluded eye blinks as a confounding cause (Jacobs et al., 2014).

We also previously showed that TMS pulses can suppress subsequent orientation stimuli (horizontal or vertical bars) (de Graaf et al., 2011), and recently demonstrated that this effect was spatially unspecific for an SOA of −50 ms, analogous to the findings for symbolic stimuli (Jacobs et al., 2014). Interestingly, with TMS pulses applied at SOA −20 ms, we moreover observed a local, spatially specific suppression effect, which was not observed with symbolic stimuli. While these results suggested two distinct mechanisms involved in orientation processing, one spatially specific and one unspecific, that experiment did not include a chronometric design and control for eye blinks. It would be meaningful to chart the spatiotemporal relevance of EVC for subsequent orientation processing in more detail.

In the current work, we thus assessed chronometrically, and with control for both eye blinks and partial pupil covering, the contribution of EVC to orientation processing. We included both objective (measured by a forced-choice orientation discrimination task) and subjective (measured by an orientation visibility rating scale) measures of vision. Our results will show that pre-stimulus TMS pulses can suppress orientation processing both locally (spatially specifically) and in a spatially unspecific manner, also after removal of trials with eye blinks or partial pupil covering, and we present the time course of both effects.

## Methods

### Participants

Twenty three volunteers came to the TMS lab for the current study. Two could not participate due to contraindications for TMS during safety pre-screening. Nine participants could not be included because they did not perceive reliable phosphenes, either at all or in the correct visual field location. They were therefore never tested in the main experiment. While it may not necessarily be the case that TMS masking is ineffective in participants without phosphene perception, the localization procedure using phosphenes ensured that the TMS coil was positioned such that the exact lower-left (LL) stimulus location was targeted and not the upper-right (UR) location (see below). Twelve participants satisfied screening and phosphene localization criteria and were tested in the main experiment. All of these participants (nine females, two authors (T.G., M.F.), nine fully naïve) were also included in the analyses and results reported here. Two participants were tested twice, one after having been subject in a preceding pilot measurement (T.G.), while one was retested because after the first measurement it was ascertained that stimulus parameters had been based on incorrect calibration data. For both subjects, only the retest results were included in this report. Participants had (corrected to) normal vision and received monetary compensation for their time. The experiment was approved by the local ethical committee.

### Stimuli, task, design

**Figure 1:**
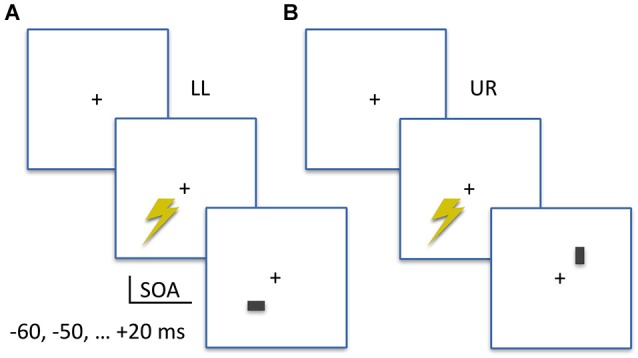
**Experimental Design**. **(A)** Example trial of the lower-left (LL) condition. The TMS pulse is applied to LL visual field location, at an SOA prior to (−60, −50, −40, −30, −20, −10 ms) or after (+20 ms) visual stimulus onset. A visual stimulus (horizontal or vertical) appears in the TMS-targeted lower-left location (LL). Participants first report the orientation of the bar, then rate the subjective visibility of the orientation. **(B)** Example trial of the upper-right (UR) condition. Same stimuli and SOAs as in the LL condition, but visual stimuli appear in the UR location.

As shown in Figure 1, stimuli were either horizontal or vertical bars (7 by 12 pixels; approximately 0.2 by 0.3 visual degrees) presented for two frames (33.4 ms) at 4 degrees eccentricity diagonally either to the LL or the UR of a central fixation cross, at a viewing distance of 57 cm. All stimuli were presented on a Iiyama ProLite monitor set to 60 Hz refresh rate and 1280 by 1024 pixel video mode in Presentation Software (Neurobehavioral Systems, Albany, CA, USA). Stimulus brightness was different across participants, as determined in a prior stimulus calibration measurement (see below), and presented on a uniform gray background. Participants performed a two-alternative forced-choice orientation discrimination task, indicating always by keyboard button presses whether the stimulus was a vertical (left index finger, “Z” button) or horizontal (right index finger, “/” button) bar. Stimulus location (LL or UR) alternated in blocks, with written instructions on screen announcing the stimulus location for the upcoming block of trials. Each block contained 14 trials, with an equal number of horizontal or vertical bars; one for each SOA in the experiment. Within blocks, the presentation order of these trials was randomized.

The SOAs implemented in this chronometric design were −60, −50, −40, −30, −20, −10 and +20 milliseconds (ms). The negative SOAs indicate that TMS pulses were administered prior to visual stimulus appearance on screen. Separately for both stimulus locations on screen, a prior monitor calibration measurement using an oscilloscope linked to the parallel port (sending TMS triggers) and a photodiode attached to the monitor (displaying visual stimuli) determined location-specific monitor delays. These were used to adapt the TMS trigger timing, to ensure SOAs as reported above with millisecond precision. With 18 trials per condition cell, in a SOA (seven levels) × StimLoc (stimulus location; two levels) within-subjects design, a grand total of 252 trials were obtained. We implemented a jittered inter-trial interval around 7 s on average (6, 7, or 8 s), which means when including breaks that the TMS measurement took a little over 30 min.

Prior to the actual TMS measurement, participants were informed, screened for TMS safety, and tested for phosphene perception. If they could be included, they next performed a stimulus calibration measurement. They wore ear plugs to protect hearing. During the active TMS measurement, we concurrently recorded eyetracking data.

### Stimulus calibration and sham TMS

The stimulus calibration measurement was included for three reasons: To ensure equal difficulty of the perceptual task across participants, to ensure equal difficulty across the two stimulus locations, and to serve as a limited form of SHAM TMS (this will be explained below).

The stimulus calibration measurement consisted of 324 trials, in blocks of LL and UR stimuli. Blocks were always announced and stimuli were horizontal or vertical bars (with orientation randomly selected per trial). Importantly, stimuli were principally black on a gray (RGB 155,155,155) background, but superimposed with a fully covering patch of the same background color. The transparency of this patch is denoted by “alpha”, where alpha ranges from 0 (full transparency; black stimulus is visible) to 255 (no transparency; black stimulus is invisible). Effectively, this results in a range of luminances and thus contrast for the stimulus. Stimuli were presented at nine levels of luminance/contrast, from alpha 210 to 250 in steps of five. Separately for both stimulus locations, 18 trials were presented per luminance/contrast level. Upon completion of this calibration measurement, a custom script in MATLAB (The MathWorks, Inc., Natick, MA, USA) was used to immediately analyze results. For the stimuli in the main experiment, we extracted per participant and per stimulus location the highest alpha value (most difficult stimulus) still resulting in correct orientation discrimination in more than 88% of trials, minus alpha 10. Thus, we made the stimulus slightly easier to perceive since prior experience dictated that participants generally perform worse in the actual TMS experiment (presumably due to somatosensory presence of TMS pulses, longer and less predictable inter-trial intervals, and perhaps increased fatigue).

While the calibration procedure did not result in physically identical orientation stimuli across participants, or between stimulus locations within participants, *post hoc* analysis showed that on average the selected alpha values were not significantly different between stimulus locations (mean (standard deviation) of alpha values for LL: 227.1 (5.0), and UR: 227.9 (5.8), not different: *t*_(11)_ = −0.518; *p* = 0.615). But more importantly, accuracy on the orientation discrimination task during calibration was indeed not significantly different between LL and UR stimulus locations for either the selected alpha values (proportion correct for LL: 0.98 (0.04), UR: 0.99 (0.03), not different: *t*_(11)_ = −0.804; *p* = 0.438) or the initial alpha values satisfying the >88% correct criterion (LL: 0.93 (0.04), UR: 0.94 (0.04), not different: *t*_(11)_ = −1.393; *p* = 0.191). In sum, stimulus luminance/contrast and associated task difficulty were customized for each stimulus location for each participant such that task difficulty was equal between stimulus locations.

**Figure 2:**
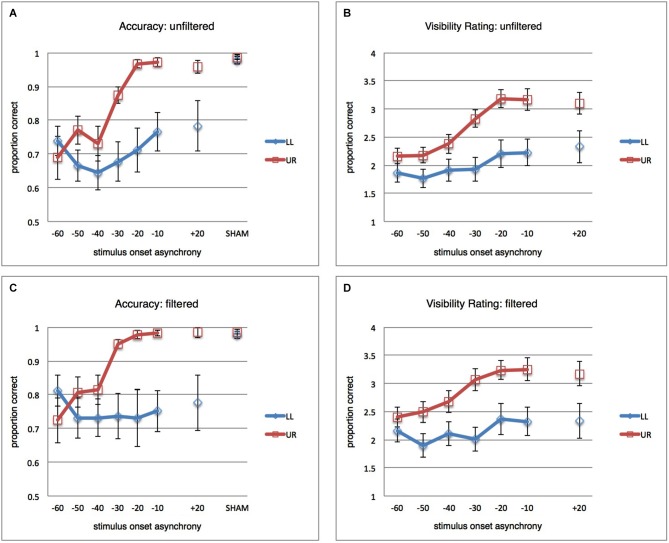
**Main Results**. **(A)** Mean proportions correct (vertical axis) over participants (*n* = 12), error bars reflect standard error of the mean (SEM). Blue curve reflects results for the LL condition (stimuli presented in the lower-left, TMS-targeted, location). Red curve reflects results for the UR condition (stimuli presented in upper-right location). TMS pulse-visual stimulus onset asynchronies (SOAs) are on the horizontal axis. Results from the calibration/SHAM measurement (see Section Methods) are provided as well, for context. As discussed in the main text, the most important observation is that TMS disrupts visual performance spatially non-specifically for the earliest SOAs (−60, −50, −40 ms) and spatially specifically for the later SOAs (−30, −20, −10 ms). **(B)** Average ratings of stimulus orientation visibility (subjective report on a 4-point scale) over participants (*n* = 12). **(C)** Same as in **(A)**, but for filtered data: All trials removed that had any indication of eye blink or partial pupil covering in a conservative window of −200 to +300 ms from visual stimulus onset. **(D)** Same as in **(B)**, but for filtered data.

During calibration, each trial contained not only visual stimulation but also a SHAM TMS pulse. SHAM TMS was achieved by tilting the TMS coil to hold it perpendicularly to occipital cortex, SOA was randomly selected per trial from −50, −40, −30, −20 ms. This made the stimulation calibration phase as much as possible comparable to the actual TMS experiment, and moreover allowed the calibration data for the eventually selected stimuli to serve as a form of SHAM baseline for comparison with the subsequent TMS experiment (18 trials as in active TMS SOA condition cells). In the remainder of this article, when referring to calibration/SHAM, these are the data we refer to. It is not a perfect baseline, since SHAM trials were not randomly interleaved with active TMS trials, inter-trial interval in the stimulus calibration measurement was much shorter than in the active TMS measurement (around 2 s, depending on subject response time), and since intensity for SHAM TMS was set to 65% (as opposed to 70%) to avoid coil overheating with this shorter inter-trial interval. Yet we have previously established that no implementation of SHAM TMS is a perfect baseline (Duecker and Sack, 2013) but that, irrespectively, SHAM TMS can rather closely approach active TMS as a control condition when it comes to controlling for non-specific TMS effects (Duecker et al., 2013). Most importantly, the hypotheses and conclusions of the current study do not actually rely on contrasts with the calibration/SHAM data. The calibration/SHAM results shown in Figure 2 serve purely as context and not included in any statistical analyses.

### TMS parameters and phosphene localization

TMS pulses were administered with a figure of eight coil (MC-B70) connected to a MagPro X100 stimulator (MagVenture, Farum, Denmark). The coil handle was pointed laterally to the right, and all participants were stimulated over right EVC, meaning occipital cortex to the UR of the inion. Thus, the LL visual field stimulus location was targeted by TMS pulses. This means that any local TMS effect should suppress stimuli presented at LL location, and not at the UR location. Spatially unspecific masking effects would suppress both stimulus locations. Intensity of TMS was fixed to 70% of machine output as in Jacobs et al. (2014), except for one participant who found this overly uncomfortable. Since this participant perceived phosphenes at a relatively low intensity we judged that stimulation at 65% would be acceptable, which was acceptable also to the participant. We here selected a fixed intensity for practical reasons, please note that it may not be the best strategy for TMS studies generally. Overall, TMS was well-tolerated by all participants.

Prior to the active TMS measurement we determined whether participants could perceive phosphenes at all, and if so, whether we could successfully elicit phosphenes in the LL visual field overlapping with the LL stimulus location at 4 degrees eccentricity. Only participants who satisfied this criterion were subsequently tested, which amounted to 12 out of 21 volunteers in the current study. We fixed the TMS coil in a mechanical coilholder, positioned such that phosphenes were elicited in the desired visual field location. Previous research showed that phosphenes overlap topographically with TMS-induced scotomas in the majority of participants (Kammer, 1999).

### Eyetracking

We performed high-fidelity eyetracking using the Eyelink1000 system (SR Research, Mississauga, Ontario, Canada), with 1000 Hz temporal resolution, to identify trials with eye blinks or pupil covering. This system automatically flags onsets and offsets of eye blinks, by the loss and recapture of pupil reflection. The pupil reflection of arbitrarily one eye was tracked. Using custom scripts in Matlab (The MathWorks, Inc., Natick, MA, USA) we checked a window of 200 ms before until 300 ms after visual stimulus onset for the presence of eye blinks, and labeled trials as “blink-trials” if this window included an eye blink flag. Behavioral results were analyzed both including and excluding these blink-trials. Secondly, we used custom Matlab scripts to identify patterns of eyetracking signal indicative of partial pupil covering. Partial pupil covering, where the pupillary reflection was not eradicated completely so that no “blink flags” were marked, might still lead to decreased visual perception. Such incomplete pupil covering can nevertheless be determined from the eyetracking data by detection of an abrupt change in recorded pupil size. To define pupil covering, we computed a threshold based on the median and standard deviation of the pupil size change over time for each trial, analogous to microsaccade detection procedures described elsewhere (Engbert and Kliegl, 2003). Specifically, we excluded trials where the change of pupil size exceeded the median value by four times the standard deviation for at least 10 ms. Thus, “filtered data” below will refer to data in which all trials were excluded that contained either blinks or signs of partial pupil covering, in the window of −200 to +300 ms from visual stimulus onset.

For one subject that otherwise seemed to hardly blink, we noticed that the eyetracker unfortunately refocused on our chinrest, as opposed to the pupil, for a significant portion of the measurement session. This was obviously recorded as a lack of pupil signal, and therefore fell in the category of “blink trial” and was filtered out in the analysis without blinks. This illustrates how conservative the filtering process was: only trials with a consistent and stable pupil signal remained. For details about the proportion of data thus excluded, see Section Results.

### Analyses

Trials with response patterns going against instruction (e.g., missing a response, pressing wrong buttons, or buttons in the wrong order, etcetera) were excluded: 1.1% of all trials. On remaining data, General Linear Model Repeated-Measures Analyses of Variance (RM-ANOVAs) were used to analyze behavioral data, both for the objective two-alternative forced-choice orientation discrimination task (mean proportion of trials with correct orientation response, per condition cell) and the subjective orientation visibility rating task (on a scale of 1 to 4, mean rating per condition cell). In follow-up analyses, RM-ANOVAs were used when indicated, and Holm-Bonferroni-corrected one-tailed *t*-tests were used (Holm, 1979). All analyses were performed using SPSS statistical software (IBM, Armonk, NY, USA), and *t*-test results were double-checked with results from non-parametric Wilcoxon signed rank tests (not reported). Results for RM-ANOVAs are presented with degrees of freedom Greenhouse-Geisser corrected if the sphericity assumption was violated according to Mauchly’s test.

## Results

### Orientation discrimination: unfiltered results

A repeated-measures ANOVA (RM-ANOVA) on proportions correct with factors SOA (seven levels) and stimulus location (StimLoc) revealed main effects of both SOA (*F*_(2.427,26.694)_ = 9.497; *p* < 0.001; *η*^2^=0.463) and StimLoc (*F*_(1,11)_ = 8.801; *p* = 0.013; *η*^2^ = 0.444). More importantly, there was an interaction between the two (*F*_(2.898,31.882)_ = 5.437; *p* = 0.004; *η*^2^ = 0.331). This reflects the observation in Figure 2A that masking of LL orientation stimuli occurs for all SOAs (confirmed by lack of main effect of SOA in follow-up RM-ANOVA for LL: *F*_(2.377,26.147)_ = 2.169; *p* = 0.127; *η*^2^ = 0.165), while UR stimuli were masked only at earlier SOAs (main effect of SOA in follow-up RM-ANOVA for UR: *F*_(2.471,27.177)_ = 15.167; *p* < 0.001; *η*^2^ = 0.580). It is interesting that UR stimuli should be masked at all, considering that TMS spatially targeted only LL stimuli. From Figure 2A it indeed becomes clear that spatially unspecific masking (orientation discrimination suppression for both UR and LL stimuli) occurred at the earliest SOAs of −60, −50, and −40 ms, but that masking of UR stimuli was no longer successful after that. In contrast, it appears that LL stimuli continued to be suppressed by later TMS pulses, suggesting that a local masking effect “kicks in” at around −30 ms. Since one of our research questions concerned the SOAs at which occipital biasing mechanisms affecting orientation processing start to work only locally, we performed pairwise comparisons of performance for both stimulus locations at each SOA (one-tailed *t*-tests, Holm-Bonferroni corrected for multiple comparisons). Accuracy was significantly lower for LL stimuli when TMS pulses were administered at −30, −20, and −10 ms (corrected *p*’s < 0.05). Non-parametric tests yielded similar results. Thus, TMS pulses at negative SOAs could suppress orientation discrimination performance both across the visual field (−60, −50, −40 ms) or only in the retinotopically targeted location (−30, −20, −10 ms). For context, discrimination performance in calibration/SHAM TMS (see Section Methods) is also displayed in Figure 2A.

### Orientation discrimination: filtered results

We reanalyzed the behavioral results after removing trials with eye blinks or partial pupil covering (see Section Methods), where the window in which we checked for any sign of blinks or pupil covering was conservatively large (from 200 ms prior to visual stimulus up to 300 ms after stimulus). This resulted in exclusion of 31.8% of all trials. Results are shown in Figure 2C.

The full-model RM-ANOVA revealed again the main effects (SOA: *F*_(6,66)_ = 3.581; *p* = 0.004; *η*^2^ = 0.246, StimLoc: *F*_(1,11)_ = 7.955; *p* = 0.017; *η*^2^ = 0.420), and importantly the interaction (*F*_(6,66)_ = 5.779; *p* < 0.001; *η*^2^ = 0.344). Again masking appeared uniform across SOAs for stimuli at the LL (RM-ANOVA with factor SOA: *F*_(6,66)_ = 0.546; *p* = 0.771; *η*^2^ = 0.047) while it was dependent on SOA for stimuli at the UR (*F*_(2.063,22.696)_ = 12.498; *p* < 0.001; *η*^2^ = 0.532). Follow-up one-sided Holm-Bonferroni corrected *t*-tests between both stimulus locations per SOA revealed largely the same statistical pattern as described above for unfiltered data. Holm-Bonferroni corrected one-tailed *t*-tests revealed differences at SOAs −30 ms, −20 ms, and −10 ms between performance at the two stimulus locations. Nonparametric tests supported this pattern. These data essentially suggest that blinks did not to any meaningful extent determine the suppression effects. As in the unfiltered dataset, results after filtering out trials with eye blinks or partial pupil covering suggested a pattern of spatially unspecific masking at early SOAs (around −60, −50, −40 ms) and local, retinotopically specific, masking at later SOAs (around −30, −20, −10 ms).

### The influence of eye blinks on performance in the current study

To further evaluate the influence of blinks on absolute levels of performance, we subtracted per participant per condition cell (SOA × StimLoc) the mean unfiltered performance from the mean blink-filtered performance. Across subjects and conditions, the absolute mean increase in accuracy due to blink-filtering was moderate, with an increase of 0.040 in proportion of correct trials. The difference was, as should be expected, a bit larger for the earlier SOAs where spatially unspecific suppression occurred (0.064 for −60, −50, −40 ms, vs. 0.027 for −30, −20, −10 ms). But this difference can be considered moderate, especially taking into account that eye blinks have sometimes been suggested to fully explain (unspecific) pre-stimulus masking effects, and considering that our conservative exclusion criteria removed quite a few trials.

In a *post hoc* analysis, we excluded the two subjects with most eye blinks and fewer than ten trials per condition cell on average after filtering (although one of these two was the subject where the eyetracker had refocused on the chinrest for a portion of the measurement, see Section Methods, and probably hadn’t in fact blinked very often). For this subject sample of ten participants, the total proportion of trials filtered out due to eye blinks or pupil covering was 25.5%, and RM-ANOVA still yielded the relevant StimLoc × SOA interaction (*F*_(6,54)_ = 5.979; *p* < 0.001; *η*^2^ = 0.399).

### Subjective ratings

Participants were asked, on each trial, to subjectively rate the visibility of the stimulus orientation, on a 4-point scale (one meaning “no perceived orientation”, four meaning “clearly perceived orientation”) (see, e.g., Overgaard et al., 2004). Results are displayed in Figure 2B and can be observed to generally follow objective discrimination performance.

All trials included, the RM-ANOVA yielded main effects (SOA: *F*_(1.574,17.314)_ = 14.682; *p* < 0.001; *η*^2^ = 0.572, StimLoc: *F*_(1,11)_ = 17.093; *p* = 0.002; *η*^2^ = 0.608), and an interaction (*F*_(2.648,29.126)_ = 8.013; *p* = 0.001; *η*^2^ = 0.421). Follow-up RM-ANOVAs revealed effects of SOA for both locations (LL: *F*_(1.896,20.855)_ = 4.288; *p* = 0.029; *η*^2^ = 0.281, UR: *F*_(1.732,19.047)_ = 23.069; *p* < 0.001; *η*^2^ = 0.677). Note that this differs from the accuracy results, in that no main effect of SOA reached significance there for stimuli presented LL of fixation. This pattern demonstrates even more clearly that masking occurred for both stimulus locations, but with different patterns over SOAs. Holm-Bonferroni-corrected one-tailed *t*-tests, however, revealed significantly lower visibility ratings for LL stimuli at all SOAs. In the blocks with LL stimuli, subjective visibility ratings were therefore consistently lower, as compared to ratings in UR stimulus blocks. We think it prudent not to interpret this difference as a fundamental finding about the brain or differential roles for pre-stimulus mechanisms in objective vs. subjective vision. Rather, it seems likely that this difference between both measures is a methodological issue, related to response tendencies. The subjective ratings are difficult because ambiguous, and—as revealed by the orientation discrimination results—there was overall more suppression in the LL stimulus blocks. It is plausible, therefore, that participants would generally provide lower ratings during the LL blocks as a whole, thus also for the earliest SOAs, simply because those were the blocks where they quickly learned that stimuli were less often perceived. We will not further consider this difference between objective and subjective results.

Removing trials with eye blinks and pupil covering, the RM-ANOVA yielded the same pattern of results (SOA: *F*_(1.754,19.290)_ = 11.215; *p* = 0.001; *η*^2^ = 0.505, StimLoc: *F*_(1,11)_ = 21.217; *p* = 0.001; *η*^2^ = 0.659, SOA × StimLoc: *F*_(3.309,36.395)_ = 4.893; *p* = 0.005; *η*^2^ = 0.308). Separate RM-ANOVAs resulted in a statistical trend for SOA for LL (*F*_(2.325,25.577)_ = 2.773; *p* = 0.074) and a significant effect of SOA for UR (*F*_(1.914,21.053)_ = 17.892; *p* < 0.001; *η*^2^ = 0.619). Holm-Bonferroni corrected one-tailed SOA-specific *t*-tests between stimulus locations revealed lower visibility ratings at all SOAs except −60 ms. On the whole, the pattern of statistical outcomes and results depicted in Figure 2D seem in line with the orientation discrimination results, aside from the overall lower ratings for LL as opposed to UR in specifically the subjective measure. As was the case for the orientation discrimination results, removing trials with eye blinks did not strongly affect or abolish the findings observed with unfiltered data.

### *Post hoc* analysis: +20 ms

In spite of several reports in the literature (see Section Discussion), based on our own previous experiences we did not anticipate masking effects in the +20 ms SOA and thus included this as an additional baseline measure. However, the results displayed in Figure 2 do suggest that spatially specific suppression occurred at +20 ms in the current experiment. This difference did not survive statistical thresholds (see above), perhaps because, as closer inspection of the data suggested, the difference between LL and UR performance at +20 ms was based nearly exclusively on four subjects out of our total twelve. To judge whether our pre-stimulus effects may be distinct from a potential +20 ms effect, we therefore decided to do additional *post hoc* analyses. Note that the following analyses are exploratory and outcomes therefore preliminary. We explored to what extent our data support the pre-stimulus pattern of results (i.e., a StimLoc by SOA interaction) after taking into account the unexpected suppression effect at +20 ms.

One way we addressed whether the pre-stimulus TMS effects were mediated by a suppression effect at +20 ms was to include the magnitude of the latter as covariate in a RM-ANCOVA on the remaining data, evaluating whether the main finding of a StimLoc(2) × SOA(6) interaction still held. Thus, we subtracted per participant the difference between accuracy at the LL stimulus location from accuracy at the UR stimulus location for the +20 ms SOA. Entering the resulting vector as covariate in the RM-ANCOVA did not yield a significant interaction of the covariate with the StimLoc × SOA interaction term, suggesting that the StimLoc × SOA interaction effect does not depend on the magnitude of masking at +20 ms. Nevertheless, not centering the covariate (since we were interested in pre-stimulus effects in the absence of +20 ms suppression) did strongly reduce the statistical power of the StimLoc × SOA interaction in the model, depending on the dataset evaluated. The interaction remained significant for orientation discrimination performance for the filtered data (*F*_(5,50)_ = 2.652; *P* = 0.033; *η*^2^ = 0.210), approached significance for the unfiltered data (*F*_(2.988,29.877)_ = 2.823; *P* = 0.056; *η*^2^ = 0.220), approached significance for unfiltered subjective ratings (*F*_(5,50)_ = 2.358; *P* = 0.054; *η*^2^ = 0.191), though significance was not reached for filtered subjective ratings.

**Figure 3:**
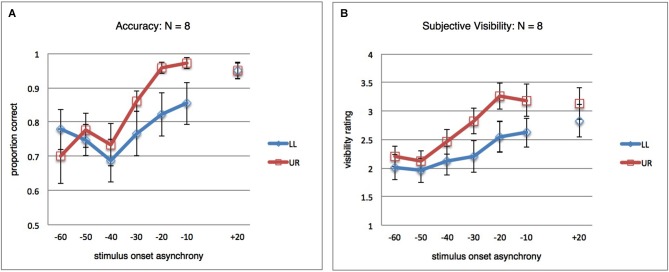
**Results for *post hoc* analysis. (A)** Orientation discrimination accuracy, as in Figure 2A, but for a subset of participants (*N* = 8). The four participants excluded here were responsible for the difference between LL and UR at SOA +20 ms observed in Figure 2A. In the remaining subject sample, the pattern of global and local masking mirrors that observed in the full dataset. **(B)** Same as in **(A)**, but results for the subjective orientation visibility rating task.

As a secondary follow-up analysis, we thought it was interesting to look at the results after exclusion of the four participants responsible for the apparent local suppression at +20 ms. The unfiltered results for the remaining eight participants are shown in Figure 3A, where clearly no difference remains between both stimulus locations at +20 ms. A repeated-measures ANOVA (SOA(7) × StimLoc(2)) on the subsample data still revealed an interaction (*F*_(6,42)_ = 2.662; *p* = 0.028; *η*^2^ = 0.276). For subjective visibility ratings, the analogous results are shown in Figure 3B, again a significant interaction (*F*_(6,42)_ = 4.812; *p* = 0.001; *η*^2^ = 0.407) (not shown; analogous analyses for filtered results also yielded significant interactions for both objective and subjective data).

Altogether, these exploratory *post hoc* results provide some suggestive, though inconclusive, support for the idea that our pre-stimulus data patterns may exist independently of a putative suppression effect at +20 ms. Future studies might address in more depth whether there is at the population level indeed a suppression effect at +20 ms within our parameters, what underlies the observed interindividual variability, and whether the spatially specific and unspecific pre-stimulus suppression effects are elicited by a different neuronal mechanism or not.

## Discussion

In this study we explored the functional relevance of EVC, *prior to* visual inputs, for orientation processing in a chronometric design, moreover controlling for eye blinks and partial pupil covering. TMS pulses were administered to occipital cortex in several SOAs to disrupt, or bias, neuronal mechanisms before horizontal or vertical bars were presented on screen. Participants performed a forced-choice discrimination task, and provided a subjective visibility rating on each trial. Both of these objective and subjective measures of orientation processing were impaired by preceding TMS pulses. Moreover, the temporal pattern of suppression of stimuli at the TMS-targeted visual field location (LL) and at a non-targeted visual field location (UR) demonstrated that TMS pulses can impair orientation processing not only locally, but also in a spatially unspecific manner. Thus, two distinct processes seem to bias subsequent orientation processing with different spatiotemporal profiles. Importantly, removal of all trials with eye blinks in a conservatively wide window around visual stimulus presentation as well as removal of trials in which the pupil was partly covered by eyelids did not abolish these results, providing further evidence for a neuronal basis of these behavioral effects.

### The SOA +20 ms

The results at the SOA of +20 ms, originally intended as an extra control condition (see for example, Camprodon et al., 2010), are interesting. The results figures may suggest that some local masking (specific to LL stimuli) occurred at this SOA, even if conservative statistical contrasts did not reach significance. It has previously been suggested that visual stimuli *can* be masked by TMS pulses at SOAs around +20 to +40 ms (Corthout et al., 1999a,b, 2002, 2003), depending on stimulus parameters (Paulus et al., 1999), but we ourselves did not observe such a masking effect in our previous work (Sack et al., 2009; Jacobs et al., 2012a). This elusive masking window may be particularly sensitive to experimental parameters, or specific to participants (Kammer et al., 2003), and indeed close inspection of individual participant data in the current subject sample revealed that the difference in performance between LL and UR stimuli (thus, the local masking effect) was nearly exclusively caused by four participants with very low discrimination performance at this SOA. These participants did also display strong masking at the other SOAs, yet *post hoc* analyses (see Section Results) provided preliminary indication that at least the main finding of a stimulus location by SOA interaction may not be fully tied to, or dependent on, the masking effect at +20 ms. The combination of this subject-specificity with the lack of a Holm-Bonferroni-corrected statistically significant effect of stimulus location at +20 ms leads us to not speculate on this SOA further at this point, as it is beyond the scope of this article. However, this was the first time we included this SOA in an experiment with simple static stimuli which were achromatic (Paulus et al., 1999). Since masking in this SOA is not always observed, the current results in this respect warrant some consideration and may inspire further investigation.

### Two mechanisms of pre-stimulus masking

In line with pioneering work by Corthout et al. (1999a,b, 2000, 2003), we recently obtained results suggesting the existence of two mechanisms biasing subsequent visual perception, using similar orientation stimuli (Experiment 1 in Jacobs et al., 2014). Yet, in that particular study only the global mechanism could be replicated when using more complex arrow stimuli and a chronometric experimental design. In the current study, we implemented such a chronometric design, yet replicated and extended the two mechanisms suggested by our earlier work, moreover controlling for eye blinks and pupil covering. Taking these results together with our previous work, this suggests that the local pre-stimulus masking effect may be either specific to orientation stimuli, or otherwise sensitive to basic stimulus parameters.

While the current dataset strongly supports the existence of local orientation suppression at several SOAs prior to visual stimulation, we cannot know with certainty how far this local masking extends temporally. Global masking occurs at −40, −50 ms and earlier, causing performance to be so low it may hide concurrent local masking effects. So whether or not local mechanisms biasing subsequent orientation processing efficacy exist prior to −30 ms remains unknown. The results do suggest that the local mechanism extends at the very least from immediately prior to visual stimulus presentation backwards in time to the onset of the global suppression mechanism, which was around −40 ms.

### Possible mechanisms underlying the spatially specific effect

The neuronal processes underlying the local biasing mechanism remain unclear. Our analyses and the spatial specificity of the effect rule out influences of eye blinks, partial pupil covering, and general attentional effects, suggesting a local neural process. The TMS pulse seems to induce a local unfavorable brain state, in other words disrupting a cortically and functionally local mechanism reflecting visual state-dependency. We previously (Jacobs et al., 2014) noted that evidence from motor cortex studies suggests single TMS pulses can have longer-lasting inhibitory and facilitatory effects (Chen, 2004) on neural circuitry. Moliadze et al. (2003) moreover demonstrated that single TMS pulses have lasting effects on visual cortex neurons as well. For example, strong TMS pulses led to a phase of inhibited neuronal activity for up to 100–200 ms. Perhaps the local pre-stimulus TMS pulse indeed instigates a temporal pattern of facilitatory/inhibitory phases in local cortical circuitry, such that the eventual arrival of visual inputs tens of milliseconds later falls into an unfavorable excitability phase.

There has also recently been renewed interest in the connection between TMS masking and visual masking, where backwards (metacontrast) visual masking (a visual mask presented after a visual target) could correspond to post-stimulus TMS masking, while forward (paracontrast) visual masking (a visual mask presented prior to a visual target) might correspond to pre-stimulus TMS masking (Tapia and Beck, 2014; see also Breitmeyer et al., 2004). Even in light of temporal commonalities, it remains unclear to what extent the neuronal mechanisms underlying these different paradigms are similar.

However, the analogy to visual masking does naturally raise a question: Is there a “visual mask” in the form of a TMS-induced phosphene? After all, TMS pulses at these intensities are, for most participants, well above phosphene threshold. That is unavoidable, since the intensities required for visual suppression are above phosphene threshold (Kastner et al., 1998; Kammer, 1999). There are at least two ways in which a phosphene could contribute to the current findings. It could either reflect local excitation, which acts as a forward mask to the subsequent visual target, or it could act on a higher-order, attentional level. Since the latter is relevant in the context of spatially unspecific masking as well, we discuss this below.

### Possible mechanisms underlying the spatially unspecific effect

Previous studies using symbolic stimuli controlled for eye blinks and other non-neural effects of TMS, showing that no pre-stimulus suppression resulted from SHAM TMS or TMS applied to a control site (vertex) (Jacobs et al., 2012a,b). We here presented evidence that also for orientation processing the spatially unspecific effect may have a neural basis. Earlier work did demonstrate that occipital TMS can induce blinks using infrared (Beckers and Hömberg, 1991; Corthout et al., 2000) or more recently high-speed video recordings (Corthout et al., 2011). But it may be difficult to compare studies in which a round coil is used to those with figure-eight coils, and the fact that blinks can be induced does not mean that blinks are necessarily the (sole) cause of suppression. Using a round coil, Corthout et al. (1999b) reported that TMS over a range of parieto-occipital scalp locations caused visual suppression, and we previously (Jacobs et al., 2014) and in the current study showed that suppression is not spatially specific. These observations, as well as the chronometry of suppression, are enough in line with eye blinks as a potential cause of suppression that this issue continues to merit consideration.

Three “artifact explanations” seem compatible with most previous findings. First, suppression is after all caused by (partial) TMS-induced pupil covering, and the employed methodologies to control for it in our studies were not sensitive enough to detect this. Ideally, a future experiment would employ high-speed video recording, of both eyes, during an ongoing pre-stimulus TMS masking study using a figure-eight coil. This would allow the most sensitive control for, and exclusion of, pupil covering on a trial-by-trial basis. Second, suppression is caused by a direct TMS-induced ocular artifact that is not pupil covering. Perhaps some muscular twitches around the eyes or small eyelid movements do affect vision in some way, even if the pupil remains uncovered. The artifact would thus not be a blocking of inputs to the retina, but a visual or attentional interference of some kind. Third, suppression is caused by an indirect TMS-induced ocular artifact. For example, though speculative, it might be that blink-responses are initiated, but inhibited before actual pupil covering, because participants are actively trying not to blink. The incomplete blink might lead to more central and neural mechanisms of suppression. Blink suppression, after all, can occur even for inputs bypassing the eyelids (Volkmann et al., 1980).

Our results are, on the other hand, also clearly in line with the hypothesis that the spatially unspecific suppression has a neural basis unrelated to ocular artifacts. The conjunction of a neural basis but a spatially unspecific consequence suggests that the functionally relevant mechanism underlying visual state-dependency affected here does not involve local circuitry. This leads to several possible explanations, which we might broadly categorize as direct vs. indirect.

A direct neural account means that the energy impulse from TMS itself has direct consequences on the cortical region representing the UR stimulus location. Thus, the stimulation would need to either “spread” to regions actively processing that visual field location, or it would need to affect a cortical mechanism that is functionally relevant for a wider visual field. With regards to the “spreading account”, it is important to note that we did not find evidence that masking was significantly weaker for the UR stimulus location, as opposed to the targeted LL stimulus location, in the early SOAs of −60, −50, −40 ms. Such a differential effect would be predicted by the most straightforward explanations in which the early (−60, −50, −40 ms) pre-stimulus suppression effect is somehow primary for the targeted visual field location but secondarily “spreads” to the other hemisphere for the opposing visual field location. One method of spreading could be through neuronal connections over the corpus callosum, a more trivial account has the induced electric field passively spread across both hemispheres at the occipital pole. A different form of spreading would be a propagation of the energy impulse to higher-order visual regions that represent a bigger part of space, encompassing both locations. Suppression could occur there, or the energy impulse might be back-projected to lower-level regions with smaller receptive fields. All these accounts seem speculative for the moment.

A different direct/neural mechanism would involve TMS disruption or excitation of an occipital mechanism that is relevant across the visual field. One candidate we have previously mentioned is a TMS-induced phase-reset, or phase-locking, of ongoing alpha oscillations in parieto-occipital regions (Rosanova et al., 2009). Previous research has shown that, given enough alpha power (Mathewson et al., 2009), alpha phase at visual stimulus onset is associated with stimulus visibility or at least visual performance (Busch et al., 2009; Mathewson et al., 2009, 2010; de Graaf et al., 2013). Recent work also linked alpha oscillatory power to global vs. local processing (Volberg et al., 2009) and repetitive TMS subsequently implicated a causal role (Romei et al., 2012). We are currently testing the possible involvement of alpha oscillations in the pre-stimulus masking effect. And there are still other oscillatory mechanisms that may be relevant to global vs. local processing, in other frequency bands (theta/beta, see e.g., Smith et al., 2006; Romei et al., 2011).

Leaving aside artifacts of sound, somatosensation, and eye blinks, there is still a potential indirect/attentional account of spatially unspecific suppression. The most prominent feature in this explanation is the likely TMS-induced phosphene, which may act as an attentional stimulus. Biasing effects through attention by the first-appearing phosphene would plausibly be spatially unspecific. And they would only appear with TMS applied to occipital cortex in this context, note the observation in Corthout et al. (1999b) that the early pre-stimulus suppression effect was observed for a range of parieto-occipital TMS coil locations. A well-known attentional phenomenon is the attentional blink (Shapiro et al., 1997; Dux and Marois, 2009; Martens and Wyble, 2010), in which detection of a first visual target impairs processing of a second visual target. It is unclear whether attentional blink, with phosphene acting as target 1 and visual stimulus as target two, is really plausible in the current paradigm, since (1) even taking into account retino-cortical delays, the interval between TMS pulse and visual target is relatively short, (2) in attentional blink paradigms there is generally a spatial correspondence between targets one and two, (3) attentional blink is much reduced when target one is ignored, and if a visual distractor does not immediately follow target 1, both of which apply here, (4) phosphenes are—particularly under conditions of low spatial attention—not very salient (e.g., Kammer et al., 2005b). Moreover, the difference in spatial attention between both stimulus locations ought to lead to a stronger phosphene for the LL position (Bestmann et al., 2007), which might predict again stronger suppression of LL stimuli.

Nevertheless, some sort of attention-grabbing by a phosphene can certainly not be excluded. Perhaps it acts as an exogenous cue that moves attention away from the UR location (Yantis and Jonides, 1996; Egeth and Yantis, 1997). It could even be that the UR suppression and LL suppression effects are mediated by different mechanisms for the same SOAs, although that may not be the most parsimonious explanation. Certainly, it will be worthwhile to attempt a pre-stimulus TMS masking study with participants that report not to perceive phosphenes. Replication of the current findings in such a subject sample would eliminate several of the proposed scenarios.

## Conclusion

Given increasing interest in how the state of the brain before or at stimulus onset influences information processing, pre-stimulus TMS masking is a paradigm with great potential. The evidence for separate early and late pre-stimulus suppression mechanisms steadily increases, although it remains unclear what the basis is for perceptual impairments in both.

## Conflict of interest statement

The authors declare that the research was conducted in the absence of any commercial or financial relationships that could be construed as a potential conflict of interest.
